# Psoriasis and Vitamin D: A Systematic Review and Meta-Analysis

**DOI:** 10.3390/nu15153387

**Published:** 2023-07-30

**Authors:** Elena Formisano, Elisa Proietti, Consuelo Borgarelli, Livia Pisciotta

**Affiliations:** 1 Department of Internal Medicine, University of Genoa, 16132 Genoa, Italy; proietti.elisa93@gmail.com (E.P.); consuelo.borgarelli@hsanmartino.it (C.B.); livia.pisciotta@unige.it (L.P.); 2 Dietetics and Clinical Nutrition Unit, IRCCS Policlinic Hospital San Martino, 16132 Genoa, Italy

**Keywords:** hypovitaminosis D, psoriasis, vitamin D supplementation, bone metabolism

## Abstract

Psoriasis is a chronic immune-dysregulated inflammatory disease and hypovitaminosis D is considered a risk factor. We conducted an online database search to review and meta-analyze the relationship between vitamin D, other bone metabolism parameters, and psoriasis. The efficacy of oral vitamin D supplementation in improving Psoriasis Area and Severity Index (PASI) was also evaluated. Non-original articles, case reports, and animal studies were excluded. Bias risk was assessed according to the Cochrane Collaboration’s tool and the Newcastle–Ottawa scale in randomized controlled trials (RCTs) and case–control studies, respectively. Unstandardized mean differences were used for data synthesis. Twenty-three studies reported serum 25 hydroxyvitamin D (25(OH)D) levels in 1876 psoriasis patients and 7532 controls. Psoriasis patients had significantly lower 25(OH)D levels than controls (21.0 ± 8.3 vs. 27.3 ± 9.8, *p* < 0.00001). Conversely, 450 psoriasis patients had lower levels of parathormone than 417 controls (38.7 ± 12.8 vs. 43.7 ± 16.5, *p* = 0.015). Four RCTs examined the effect of oral vitamin D supplementation on psoriasis for 173 patients and 160 patients were treated with placebo. No significant differences were found in PASI after 3, 6, and 12 months of supplementation. It is shown that 25(OH)D serum levels are significantly lower in psoriasis, but, although the granularity of RCT methodology may have influenced the pooled analysis, vitamin D supplementation did not seem to improve clinical manifestations.

## 1. Introduction

Psoriasis is a chronic autoimmune inflammatory skin disease, characterized by abnormal proliferation and differentiation of keratinocytes [1,2,3], which is revealed by erythematous skin plaques covered by silvery scales [4,5]. Even though the exact mechanism behind this condition is not fully understood [6], it is known that psoriatic skin lesions are the result of dysregulation of the immune system which releases pro-inflammatory mediators. Subsequently, the inflammatory response, largely driven by cytokine release, causes uncontrolled proliferation of keratinocytes [7]. The etiology of psoriasis is still unknown, but researchers have speculated about a probable complex mix of hereditary and non-hereditary factors [8], including obesity, alcohol consumption, and smoking habit [9].

The prevalence of data of psoriasis is not completely clear: it may range from 0.91 to 8.5% in adults and from 0.0 to 2.1% in children. The worldwide psoriatic disease prevalence is about 2–3% [10], with a higher amount (8–11% of the total population) in North European countries [11].

Lower serum 25 hydroxyvitamin D (25(OH)D) levels have been associated with the pathogenesis of several skin disorders, such as atopic dermatitis, vitiligo, alopecia areata, and also psoriasis [12]; in fact, vitamin D is known to influence multiple skin functions, such as proliferation, differentiation, and apoptosis of keratinocytes [13]; therefore, an abnormal vitamin D metabolism could play a key role in the pathogenesis of psoriasis [14]. Furthermore, numerous epidemiological studies indicate an association between psoriasis and a reduction in mineral bone density, suggesting an effective relationship between psoriasis and higher risk of osteoporosis and fractures [15]. Indeed, vitamin D has a central role in bone turnover, through the regulation of calcium and phosphate metabolism, and in the control of parathyroid hormone (PTH) secretion [16,17], thus suggesting to check not only the parameters directly associated with psoriasis but also those related to bone metabolism.

The severity of psoriasis can be assessed using the Psoriasis Area and Severity Index (PASI), which assigns a score that varies from 0 (absence of disease) to 72 (severe disease) based on the severity of skin manifestations [18]; moreover, the PASI score is useful in evaluating the treatment response in psoriasis patients [19]. Topical therapies, including vitamin D3 analogues, are valid treatments for mild-to-moderate psoriasis, since keratinocytes in psoriatic lesions express the vitamin D receptor [20]. Although oral vitamin D supplementation is also used [21], because of its role in immune homeostasis [22], results are still controversial [23].

Some systematic reviews and meta-analyses have evaluated the association between 25(OH)D serum levels and psoriasis, suggesting a correlation between hypovitaminosis D and psoriasis, but none of them analyzed a possible association with other parameters related to bone metabolism.

We conducted a systematic review and meta-analysis of the literature to evaluate the association of hypovitaminosis with psoriasis and to verify the efficacy of vitamin D supplementation in improving the PASI score of psoriasis patients. Our other aim was to evaluate whether serum parameters linked to bone metabolism could also be related to psoriasis.

## 2. Materials and Methods

The present systematic review and meta-analysis was conducted according to the Preferred Reporting Items for Systematic reviews and Meta-Analysis (PRISMA) statement [24,25]. PRISMA 2020 checklists are reported in Tables S1 and S2.

### 2.1. Eligibility and Inclusion Criteria

The aim of the meta-analysis was to evaluate vitamin D deficiency in patients affected by psoriasis and whether vitamin D supplementation improved the PASI score.

We included studies that met the following criteria:

- Prospective and retrospective studies in English language published as full-text articles, which included participants ≥18 years affected by psoriasis and healthy control subjects who had their 25(OH)D level measured;
- Randomized double-blind, placebo-controlled studies (RCTs) in English language published as full-text articles, which included participants ≥18 years with psoriasis who underwent placebo-controlled supplementation with ergocalciferol (vitamin D2) or cholecalciferol (vitamin D3).

Co-primary endpoints were the evaluation of vitamin D supplementation’s impact on clinical activity of psoriasis according to the PASI and the measurement of the difference in vitamin D deficiency in psoriasis patients in comparison to healthy controls. Secondary outcomes were assessing the difference in calcium, phosphorus, and PTH levels in patients with psoriasis versus controls.

### 2.2. Exclusion Criteria

We excluded articles from this meta-analysis according to the following exclusion criteria:

- Animal studies, reviews, systematic reviews, and case reports;
- Studies using topical 1,25-dihydroxycholecalciferol and topical cholecalciferol (D3);
- Studies that did not provide sufficient data on 25(OH)D levels.

### 2.3. Database Search Strategy and Selection Process

Databases were last accessed on 8 July 2023. We searched using Ovid (Embase, Scopus, and Web of Science) and PubMed, applying search the terms “vitamin D” OR “vitamin D2” OR “vitamin D3” OR “D2” OR “D3” OR “ergocalciferol” OR “cholecalciferol” OR “25-hydroxyvitamin D” AND “psoriasis”. Table S3 reports details on web research. Studies were screened by the two authors (EF and EP) and, after removing duplicate reports, were included. Institutional access and an open-access license allowed access to the full text of the included articles and any disagreements were arbitrated and resolved by discussion with a third author (LP).

### 2.4. Data Extraction and Quality Assessment

EF and EP extracted the following details: first author’s last name, year of publication, age, sex, sample size, serum 25(OH)D levels, PASI score, serum calcium, phosphorus, and PTH levels. Tables S4 and S5 report the complete dataset of the analyzed studies.

This meta-analysis was performed with studies that exclusively comprised patients with a documented psoriasis diagnosis, including psoriatic arthritis, chronic plaque psoriasis, palmoplantar psoriasis, and psoriasis vulgaris. We considered serum 25(OH)D levels measured using radioimmunoassays, enzyme-linked immunosorbent assay (ELISA) kits, competitive enzyme immunoassays, and chromatography assays.

In the RCTs, the therapeutic scheme of vitamin D administration, the form of vitamin D supplements, and the duration of supplementation were collected. We considered studies assessing the severity of psoriasis determined by the PASI score administered by a trained researcher, associate professor, or a dermatologist.

### 2.5. Bias Risk Assessment

The methodological quality of case–control studies included was evaluated by the Newcastle–Ottawa scale (NOS) criteria. Studies that have a final score of 7 or more are considered high-quality studies. Moderate-quality studies have four to six stars and poor-quality studies have zero to three stars (Tables S6 and S7). The Cochrane Collaboration’s tool for assessing risk of bias in randomized trials was used for assessing the quality of RCTs (Figure S1) [26].

### 2.6. Summary of Data and Statistical Analysis

Data were presented and analyzed using mean and standard deviation and were used to perform the meta-analysis to obtain unstandardized mean differences and 95% confidence intervals (CI). Data were pooled in a meta-analysis using RevMan 5.4.1 (the Cochrane Collaboration, Copenhagen, Denmark) and ProMeta 3 (IDoStatistics).

Statistical heterogeneity was expressed using *I*^2^ statistics. If the statistical heterogeneity (*I*^2^ > 50%) was high, a random-effects model was used to analyze data; otherwise, a fixed-effect model was preferred. Two-sided *p* values ≤  0.05 were considered statistically significant.

Egger’s regression asymmetry test and Begg’s test were used to evaluate possible publication bias, with the results considered to indicate publication bias when *p* < 0.10. The trim and fill method was used for adjustment when statistically significant bias was found. Forest and funnel plots were used to present the synthesized data and to show the assessment of the publication bias risk, respectively.

## 3. Results

### 3.1. General Characteristics of Included Studies and Patients

**Figure 1:**
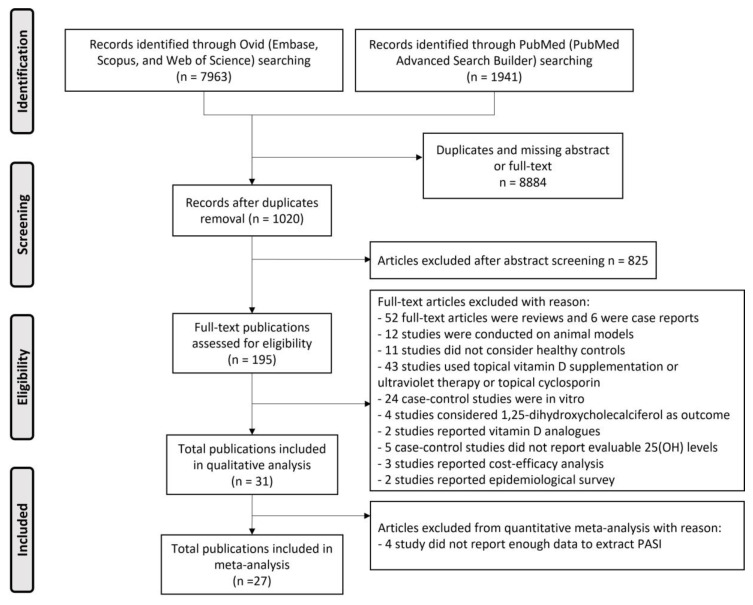
PRISMA flow chart of the literature search and selection process.

Figure 1 reports the sorting process of the publications included in the meta-analysis. A total of 18 case–control studies [27,28,29,30,31,32,33,34,35,36,37,38,39,40,41,42,43,44], 5 cross-sectional cohort studies [45,46,47,48,49], and 4 RCTs [50,51,52,53] were included, and a general description of all studies is provided in Table 1 and Table 2.

Overall, 9741 subjects were included in the meta-analysis and were allocated in two different analyses: 25(OH)D level analysis with a total of 9408 subjects (patients with psoriasis 1876 (19.9%) and healthy controls 7532 (80.1%)) and vitamin D supplement efficacy analysis in patients with psoriasis (total of 333: number of patients receiving vitamin D supplements: 173 (52.0%) and patients receiving placebo: 160 (48.0%)). Patients’ median age was 51.0 ± 5.6 and male were 4960 (50.9%).

### 3.2. Serum 25(OH)D Levels and Other Bone Mediators Involved: A Case–Control Meta-Analysis

**Figure 2:**
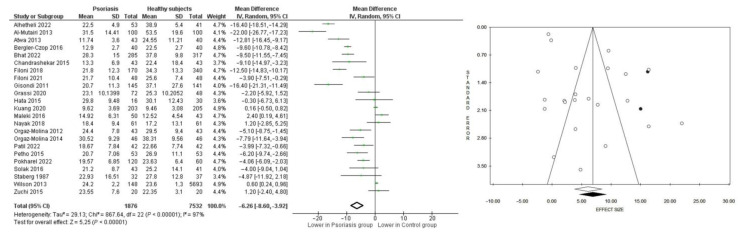
Forest and funnel plots showing mean difference for 25(OH)D levels in patients with psoriasis and healthy controls. Green squares with lines are mean differences and 95% confidence interval. Black dots are trimmed and filled studies, white dots are all studies included in the sub-analysis. White diamond is the unstandardized mean difference, while the black diamond is the unstandardized mean difference after trim and fill correction [27,28,29,30,31,32,33,34,35,36,37,38,39,40,41,42,43,44,45,46,47,48,49].

A total of 23 studies reported 25(OH)D levels in 9408 subjects, of which 1876 had psoriasis (19.9%) and 7532 were healthy controls (80.1%). When data were pooled, there was high heterogeneity (Tau^2^ = 29.13, *p* < 0.00001, *I*^2^ = 97%). Patients with psoriasis had lower 25(OH)D level than controls, resulting in a mean difference of −6.26, CI: −8.60, −3.92 ng/dL (21.0 ± 8.3 vs. 27.3 ± 9.8, *p* < 0.00001) (Figure 2). The funnel plot appeared asymmetrical; therefore, two studies were trimmed, resulting in a re-estimated mean difference of −6.92, CI: 9.34, −4.49 ng/dL, *p* < 0.00001 (*p* = 0.003 using Egger’s test and *p* = 0.731 using Begg’s test).

Seven studies reported serum calcium levels in subjects affected or not affected by psoriasis, including a total of 906 persons (468 patients with psoriasis (51.7%), 438 controls (48.3%)). There was high heterogeneity (Tau^2^ = 0.05, *p* < 0.00001, *I*^2^ = 89%) with a symmetrical funnel plot in pooled analysis with low risk of publication bias (*p* = 0.665 using Egger’s test and *p* = 0.881 using Begg’s test). However, there was no significant difference in the serum calcium levels in psoriasis patients compared to controls with a mean difference of 0.09, CI: −0.09, 0.26 mg/dL (9.6 ± 0.4 vs. 9.5 ± 0.4, *p* = 0.328).

Phosphorus serum levels were reported in four studies, thus including 392 patients, of which 209 had psoriasis (53.3%) and 183 were healthy controls (46.7%). The heterogeneity was low (Chi^2^ = 3.27, *I*^2^ 8%) with a symmetrical funnel plot in pooled analysis and low risk of publication bias (*p* = 0.679 using Egger’s test and *p* = 0.497 using Begg’s test). The phosphorus serum levels between the two groups did not differ significantly (mean difference of −0.04, CI: −0.15, 0.08 mg/dL; 3.4 ± 0.5 vs. 3.5 ± 0.6, *p* = 0.510).

**Figure 3:**
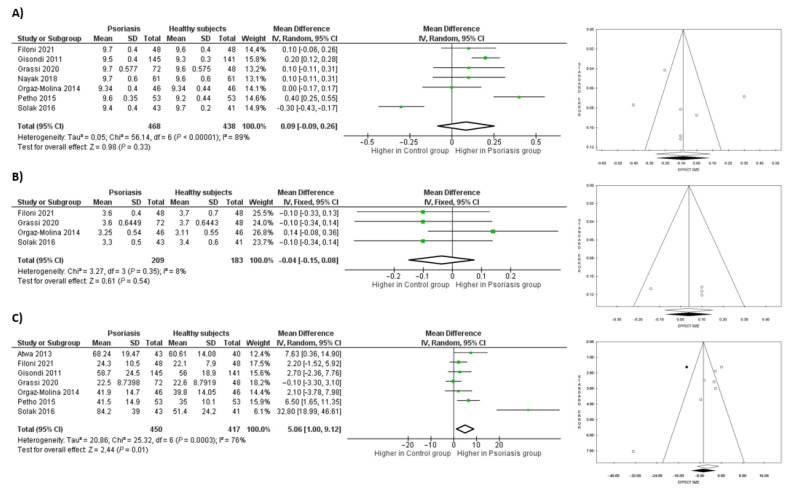
(**A**) Forest and Funnel plots showing mean difference for (**A**) Calcium levels [32,33,34,36,41,48,49], (**B**) Phosphorus levels [33,34,36,49], and (**C**) PTH levels [32,33,34,36,45,48,49] in patients with psoriasis and healthy controls. Green squares with lines are mean differences and 95% confidence interval. Black dots are trimmed and filled studies, white dots are all studies included in the sub-analysis. White diamond is the unstandardized mean difference, while the black diamond is the unstandardized mean difference after trim and fill correction.

Seven studies described serum PTH levels among 450 patients (51.9%) affected by psoriasis and 417 controls (48.1%). When data were pooled, there was high heterogeneity (Tau^2^ = 20.86, *p* = 0.0003, *I*^2^ = 76%). Patients with psoriasis had higher levels of PTH than controls, with a mean difference of 5.06, CI: 1.0, 9.1 pg/mL (43.7 ± 16.5 vs. 38.7 ± 12.8, *p* = 0.015). The Begg’s test of 25(OH)D results showed borderline significance (*p* = 0.051), while the Egger’s test showed statistical significance (*p* = 0.007) with the funnel plot presenting an asymmetrical appearance. Thus, the trim and fill method was applied and one study was trimmed, resulting in a re-estimated mean difference of 6.66, CI: 2.01, 11.31 ng/dL, *p* < 0.005. Figure 3 shows the forest and funnel plots showing mean differences for calcium levels, phosphorus levels, and PTH levels in patients with psoriasis and controls.

### 3.3. Effect of Vitamin D Supplementation on PASI

Four RCTs reported vitamin D supplementation in 333 patients affected by psoriasis, of which 173 patients (52.0%) received vitamin D supplementation and 160 patients (48.0%) received a placebo. The average time of vitamin D treatment was 8.4 months and the molecules used were vitamin D3 (*n* = 150/173; 86.7%) and vitamin D2 (*n* = 23/173; 13.3%).

PASI score was considered after 3, 6, and 12 months of vitamin D treatment. At 3 months, the pooled analysis showed low heterogeneity (Chi^2^ = 4.0, *p* = 0.26, *I*^2^ = 24%) with a mean difference of −0.03, CI: −0.42, 0.49 (2.9 ± 2.0 vs. 2.9 ± 1.8) between patients treated with vitamin D and patients receiving a placebo (*p* = 0.90). The funnel plot in pooled analysis was symmetrical and the risk of publication bias was low (*p* = 0.627 using Egger’s test and *p* = 1 using Begg’s test).

After 6 months of vitamin D supplementation, the pooled data revealed low heterogeneity (Chi^2^ = 1.132,63, *p* = 0.57, *I*^2^ = 0%) and the mean difference in PASI scores between subjects supplemented with vitamin D and controls was −0.45, CI: −1.08, 0.18 (2.5 ± 2.0 vs. 3.0 ± 1.9; *p* = 0.16). The funnel plot appeared asymmetrical; therefore, one study was trimmed, resulting in a re-estimated mean difference of −0.55, CI: −1.14, −0.04, *p* = 0.07 (*p* = 0.898 using Egger’s test and *p* = 0.602 using Begg’s test).

The PASI score after 12 months of therapy with vitamin D was reported by two out of the three studies considered. When data were pooled, there was low heterogeneity (Chi^2^ = 0.84, *p* = 0.36, *I*^2^ = 0%). Patients treated with vitamin D did not report a significant improvement in PASI score compared to subjects treated with a placebo (mean difference: 0.12, CI: −0.50, 0.74; 2.8 ± 2.3 vs. 2.7 ± 1.5; *p* = 0.70).

**Figure 4:**
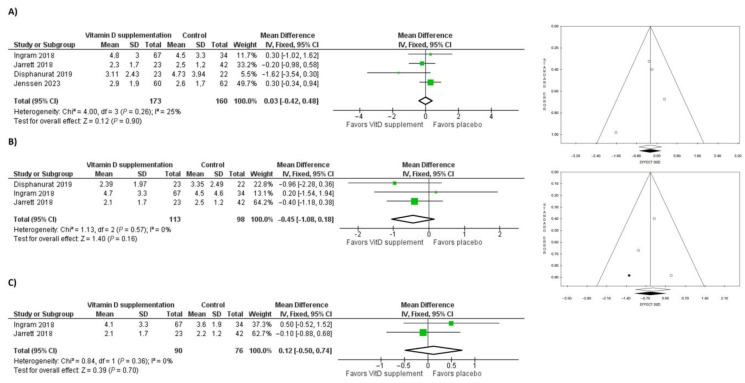
Forest and Funnel plots showing mean differences for PASI after (**A**) 3 months [50,51,52,53], (**B**) 6 months [50,51,52], and (**C**) 12 months [50,52] of vitamin D or placebo supplementation. Green squares with lines are mean differences and 95% confidence interval. Black dots are trimmed and filled studies, white dots are all studies included in the sub-analysis. White diamond is the unstandardized mean difference, while the black diamond is the unstandardized mean difference after trim and fill correction.

Forest and funnel plots showing mean differences for PASI after 3, 6, and 12 months are reported in Figure 4.

### 3.4. Risk of Bias Analysis

All case–control and cross-sectional studies included received at least seven stars in the NOS, meaning all of them were high-quality studies (Tables S4 and S5). Among the three RCTs included, one of them was at high risk of bias, while the remaining studies were at low risk of bias (Figure S1).

## 4. Discussion

This systematic review and meta-analysis of 27 studies aimed to clarify the association of hypovitaminosis D in psoriatic patients and the effectiveness of vitamin D supplementation with remission of psoriatic symptoms, according to the PASI. Subsequently, another purpose of the study was to assess the role of other serum parameters connected to bone metabolism, such as calcium, phosphorus, and PTH, in psoriatic disease.

It is known from several studies, including recent meta-analyses, that lower levels of vitamin D are commonly found in subjects suffering from autoimmune diseases such as multiple sclerosis [54], lupus erythematosus [55], and other autoimmune skin diseases [56]. Considering that psoriasis is an autoimmune disease, our study fits into this context. In fact, the results of this systematic review and meta-analysis underlined that patients diagnosed with psoriasis showed lower levels of 25(OH)D compared to healthy controls. Despite the high level of data heterogeneity, the results showed a statistically significant difference of −6.26, CI: −8.60, −3.92 ng/dL (21.0 ± 8.3vs 27.3 ± 9.8, *p* < 0.00001). Our findings were in line with a previous systematic review and meta-analysis that revealed an impacting correlation between low 25(OH)D levels and psoriasis [57]. Starting from a total of 107 articles, Pituweerakul et al. selected ten prospective cohort studies containing more than 6200 controls and nearly 700 cases to prove that people affected by psoriatic disease had a significantly reduced serum concentration of vitamin D. A statistically significant result of −6.13 ng/mL (95% CI: −10.93 to −1.32, *p* = 0.01) was obtained by comparing patients to healthy controls. Despite this, it was not possible to identify a causal relationship, which is still unknown today. Moreover, as stated by Fletcher et al. [58] and Lee YH and Song GG [59], it is still unclear if low 25(OH)D levels could constitute a consequence of psoriasis or if they represent a possible contributing factor. These latter authors are precisely those who support our conclusions. Lee YH and Song GG pointed out that the group of psoriatic subjects reported lower levels of vitamin D than the group of healthy controls (SMD = 0.64, 95% CI = 1.22 to 0.05, *p* = 0.03). A subtle but significant negative correlation between pathology severity and 25(OH)D levels was also revealed by their meta-analysis, suggesting a hypothetical role of vitamin D in the pathogenesis of this problem.

Psoriasis is an inflammatory condition where immune function appears unregulated and inflammatory cells deepen in skin wounds [60]. T-helper lymphocytes like Th1, Th17, and Th22 are recognized as the main actors of the disease, defined pathogenetically as T-cell-mediated disorder. T cells can modulate the activity of T-helper lymphocytes and immune response, especially in autoimmune-related instances [61,62]. It is in this field that vitamin D may play a role. Vitamin D is a key pro-hormone with pleiotropic effects, explained by the fact that vitamin D receptors are dispersed throughout the body. Keratinocytes, dendritic cells, macrophages, and T cells express these receptors. Other than its well-known role in calcium homeostasis, vitamin D is important in reducing inflammatory response, in balancing innate and adaptative immune response, and in keeping the cutaneous barrier intact, also through keratinocyte maturation [58]. For these reasons, a reduction in 25(OH)D levels may play a pivotal role in psoriasis pathogenesis, promoting an inflammatory general environment and involvement of the immune system, with different and local consequences, including those on the skin barrier and keratinocytes with the appearance of psoriatic lesions [63]. The reasons why vitamin D is scarce among psoriasis patients may be due to the tendency to avoid sunbathing and to abstain completely from the sun and from pharmacological therapy that can compete with its absorption. In fact, major sources of vitamin D for the human body are sun irradiation exposure and dietary adsorption; thus, a reduction in or an absence of one of them may have an impact on its deficit. Inactive forms of vitamin D such as ergocalciferol, better known as vitamin D2, and cholecalciferol, or vitamin D3, come from plants or vegetables and animal species, respectively [59,64]. The same conclusion was hypothesized by Murdaca et al. on various autoimmune diseases, including multiple sclerosis, in which low 25(OH)D levels were explained in northern countries by a reduced sun exposure and a lower consumption of vitamin D-rich foods [65].

Our investigation of other bone-related mediators focused on PTH, calcium, and phosphate. Apart from already-mentioned effects of vitamin D, this micronutrient is involved in several metabolic processes such as phosphate and calcium modulation, in addition to PTH release. This equilibrium between these micronutrients and hormones preserve bone health [16] and a vitamin D deficit can result in pathological conditions such as osteoporosis. For these reasons, we aimed to explore a possible link between psoriatic patients and other mediators pertinent to bone metabolism. Our findings regarding seven and four studies, respectively, did not reveal statistically significant differences in calcium and phosphate levels between patients diagnosed with psoriasis and healthy controls. While data from calcium were highly heterogenous, those for phosphorous were not (Chi^2^ = 3.27, *I*^2^ 8%). An interesting result was found in PTH levels: patients showed statistically increased PTH levels compared with controls: 5.06, CI: 1.0, 9.1 pg/mL (43.7 ± 16.5 vs. 38.7 ± 12.8, *p* = 0.015). Furthermore, this correlation was also found for another autoimmune disease, namely rheumatoid arthritis. Rossini et al., in their cross-sectional study, found increased PTH levels in patients with rheumatoid arthritis in comparison with healthy controls, showing a higher risk of osteoporosis [66]. A possible explanation of high PTH levels can be a relationship with vitamin D metabolism, which has been found to be reduced in psoriasis in order to maintain adequate serum calcium levels. In addition to this well-known mechanism, a different implication of high PTH levels in psoriatic patients can be speculated through results provided by Motavalli et al. [67]. They showed that high PTH levels correlate with an increased number of Th17 cells in peripheral blood. Indeed, this finding could link high PTH levels to the pathogenesis of psoriasis, in which T-helper lymphocytes 17 are involved.

Our data showed that vitamin D supplementation for a mean period of 8.4 months does not seem to be a therapeutic option for patients with psoriasis. Analyzing four RCTs, the efficacy of vitamin D supplementation was evaluated versus placebo. As previously reported by Theodoridis et al. [68], vitamin D did not show any therapeutic effect on improving the PASI score of 333 psoriatic patients after 3, 6, and 12 months of supplementation. This result is supported by Musumeci et al., who suggest vitamin D supplementation only in selected cases [69].

However, there may be other factors involved in the absorption of vitamin D, whose supplementation should be considered, as suggested by Wilchowski SM and Lareau T [70]. In fact, the authors proposed to evaluate magnesium and vitamin K2 supplementation since these supplements, acting together, could improve 25(OH)D serum levels. Furthermore, recent evidence suggested that gut microbiota seem to be increasingly connected to vitamin D metabolism [71]. Indeed, Bosman et al. hypothesized the presence of a novel skin–gut axis that could play a key role in achieving homeostasis and maintaining people’s health [72].

The lack of improvement in PASI score after vitamin D supplementation could be related to the different 25(OH)D levels achieved after the therapeutic window and, likewise, the varied methodology used in the design of included studies could play a pivotal role in such findings. In fact, a recent post-hoc analysis of the RCT conducted by Dawson-Hughes et al. suggested that a sustained plateau of 25(OH)D levels (≥100 nmol/L) can significantly reduce the risk of developing diabetes in adults with prediabetes, as compared with those who reached 25(OH)D levels between 50 and 74 nmol/L [73]. This result is inconsistent with the original analysis as no prevention of diabetes among patients with prediabetes was observed [74]. According to this evidence, it is not clear whether subjects who achieve 25(OH)D levels greater than 40 ng/mL may receive a benefit regarding their chronic diseases. This later cut-off value has also been indicated for the prevention of respiratory infection as highlighted by Grant et al. [75].

Analyzing the RCTs included in our systematic review and meta-analysis, we observed that Ingram et al. reported an increase in 25OHD levels from 24.8 ng/mL to 41.2 ng/mL with 200,000 IU followed by 100,000 IU/month of vitamin D3 [50]; a similar result was described by Jenssen et al. [53] (from 15.1 ng/mL to 29.7 ng/mL) with a 100,000 IU loading dose, followed by 20,000 IU/week, whereas Jarrett et al. reported only the baseline value (26.2 ng/mL) [52]. Instead, Disphanurat et al. showed an improvement in PASI score with 60,000 IU of vitamin D2 every 2 weeks, although 25(OH)D levels increased only from 24.77 ng/mL to 27.39 ng/mL [51]. Therefore, the heterogeneity of vitamin D supplementation is so high that a definitive conclusion is almost inconsistent. In addition, the number of studies included in our systematic review and meta-analysis is insufficient to obtain reliable conclusions.

To the best of our knowledge, this is the first meta-analysis in which not only outcomes of vitamin D were considered, but also those of phosphate, calcium, and PTH levels. Nevertheless, there are also some limitations: the limited number of studies, different study designs, and different follow-up periods. The inclusion of studies with heterogeneous methodologies (i.e., cross-sectional and case–control) was a limitation of the evaluation process, which could lead to poorly interpreted results. The difference between means was chosen for the clinician’s ease of interpretation, but it may not be superior to alternative synthesis techniques.

## 5. Conclusions

This systematic review and meta-analysis showed that vitamin D serum levels are significantly lower in patients with psoriasis than in healthy controls. However, it is still unclear if low 25(OH)D levels represent a consequence of psoriasis or a possible contributing factor. Furthermore, serum PTH levels were significantly higher in psoriatic patients than controls, suggesting a possible relationship with the pathogenesis of psoriasis. Finally, vitamin D supplementation did not significantly improve the PASI score, although vitamin D therapy has been administered to psoriasis patients for many years. It is likely that other factors are involved in the absorption of vitamin D, whose supplementation should be considered to improve 25(OH)D serum levels in patients affected by chronic diseases like psoriasis.

Further studies are needed to clarify the causal relationship between hypovitaminosis D and psoriasis and to determine the efficacy of vitamin D supplementation in patients affected by psoriasis, specifying optimal dosage, possible supplements combinations, any adverse events, and other factors involved.

## Figures and Tables

**Table 1:** Description of the included studies in the case–control and cross-sectional analysis.

Reference	Study Design	Sample Size	Psoriasis Patients	Controls	Age (Years)	Type of Psoriasis	Vitamin D Dosage	Vitamin D Deficiency—Cut Off
Bhat GH; 2022 [27]	Case–control	602	285	317	44.1 ± 13.6	NA	Chemiluminescence immunoassay	<20 ng/mL
Pokharel R; 2022 [28]	Case–control	180	120	60	42.5 ± 14.3	Patients with chronic plaque psoriasis	Chemiluminescent immunoassay	<20 ng/mL
Patil A; 2022 [29]	Case–control	84	42	42	39.7 ± 12.3	Severity of psoriasis assessed by PASI score; 35 patients presented chronic plaque psoriasis	Chemiluminescence microparticle immunoassay	<20 ng/mL
Chandrashekar L; 2015 [30]	Case–control	86	43	43	44.6 ± 12.0	Psoriasis patients with Fitzpatrick skin type V	Enzyme immunosorbent assay (ELISA) kits	NA
Zuchi MF; 2015 [31]	Case–control	40	20	20	46.4 ± 14.9	5/20 had palmoplantar psoriasis and 15/20 psoriasis vulgaris	Chemiluminescent immunoassay	<20 ng/mL
Petho 2015 [32]	Case–control	106	53	53	54.7 ± 10.5	Patients with psoriatic arthritis	Electrochemiluminescence immunoassay	<50 mmol/L
Orgaz-Molina J; 2014 [33]	Case–control	92	46	46	45.7 ± 10.0	Psoriasis patients without arthritis	Radioimmunoassay	<20 ng/mL
Solak B; 2016 [34]	Case–control	84	43	41	36.7 ± 7.8	Psoriasis patients without arthritis	NA	NA
Al-Mutairi N; 2013 [35]	Case–control	200	100	100	40.5 ± 8.8	Patients with stable plaque psoriasis (PASI ≥ 10)	Competitive enzyme immunoassay	<10 ng/mL
Filoni A; 2021 [36]	Case–control	96	48	48	50.2 ± 13.2	Patients with chronic plaque psoriasis	Direct immunometric measurement	NA
Flioni A; 2018 [37]	Case–control	510	170	340	49.4 ± 16.6	Psoriasis patients including arthropathic psoriasis	NA	<20 ng/mL
Staberg B; 1987 [38]	Case–control	69	32	37	44.0 ± 13.3	NA	Chromatography assay	NA
Hata TR; 2014 [39]	Case–control *	46	16	30	34.3 ± 11.0	Patients with mild psoriasis	NA	<20 ng/mL
Kuang Y; 2020 [40]	Case–control	408	205	203	43.7 ± 12.3	NA	Radioimmunoassay	<20 ng/mL
Nayak PB; 2018 [41]	Case–control	122	61	61	43.6 ± 3.6	Patients with chronic plaque psoriasis	NA	<20 ng/mL
Orgaz-Molina J; 2012 [42]	Case–control	86	43	43	44.1 ± 10.0	Patients with chronic plaque psoriasis	Radioimmunoassay	<20 ng/mL
Bergler-Czop B; 2016 [43]	Case–control	80	40	40	41.6 ± 14.4	Patients with moderate-to-severe psoriasis	Enzyme-linked immunosorbent assay (ELISA) kits	<20 ng/mL
Alhetheli G; 2022 [44]	Case–control	94	53	41	43.6 ± 6.0	NA	Chemiluminescence immunoassay	<12 ng/mL
Atwa M; 2013 [45]	Cross-sectional	83	43	40	47.0 ± 8.3	Patients with chronic plaque psoriasis	Chemiluminescence immunoassay	<20 ng/mL
Maleki M; 2016 [46]	Cross-sectional	93	50	43	42.1 ± 13.7	Patients with chronic plaque psoriasis for ≥10 months	Enzyme-linked immunosorbent assay	<20 ng/mL
Wilson PB; 2013 [47]	Cross-sectional	5841	148	5693	39.5 ± 5.3	NA	Radioimmunoassay	<20 ng/mL
Gisondi P; 2011 [48]	Cross-sectional	286	145	141	51.7 ± 10.2	Patients with chronic plaque psoriasis	Enzyme-linked immunosorbent assay (ELISA) kits	<20 ng/mL
Grassi T; 2020 [49]	Cross-sectional	120	72	48	50.3 ± 13.0	Patients with chronic plaque psoriasis	Chemiluminescence immunoassay	NA

* The study by Hata et al. is reported as a case–control study since we considered baseline values. Abbreviations: NA: not applicable.

**Table 2:** Description of the included studies in the Vitamin D supplementation analysis.

Reference	Study Design	Sample Size	Vitamin D Supplementation	Placebo Supplementation	Age (Years)	Type of Psoriasis	Dose	Time of Supplementation	Effectiveness
Ingram M; 2018 [50]	Randomized double-blind placebo-controlled study	101	67	34	49.4 ± 13.5	Chronic plaque psoriasis	200.000 IU of Vitamin D3 at baseline, then 100.000 IU of Vitamin D3 per month	12 months	No improvement of PASI
Disphanurat W; 2019 [51]	Randomized double-blind placebo-controlled study	45	23	22	50.9 ± 15.1	Chronic plaque psoriasis	60.000 IU of Vitamin D2 every 2 weeks	6 months	Improvement of PASI after 3 and 6 months
Jarrett P; 2018 [52]	Randomized double-blind placebo-controlled study	65	23	42	67.0 ± 8.2	NA	100.000 IU of Vitamin D3 per month	12 months	No improvement of PASI
Jenssen M; 2023 [53]	Randomized double-blind placebo-controlled study	122	60	62	53.7 ± 10.0	Active plaque psoriasis	100.000 IU of Vitamin D3 at baseline, then 20.000 IU of Vitamin D3 per week	4 months	No improvement of PASI

Abbreviations: PASI: Psoriasis Area and Severity Index.

## Data Availability

All data were available in the study. No new data were created in this study.

## Supplementary Materials

The following supporting information can be downloaded at: https://www.mdpi.com/article/10.3390/nu15153387/s1, Table S1: PRISMA 2020 for Abstracts checklist; Table S2: PRISMA 2020 checklist; Table S3: Search strategy of electronic databases; Table S4: Complete dataset for the case–control and cross-sectional analysis; Table S5: Complete dataset for the randomized controlled studies included in Vitamin D supplementation analysis; Table S6: Quality scoring for included 14 articles using Newcastle–Ottawa Scale (NOS) for case–control studies; Table S7: Quality scoring for included 4 articles using Newcastle–Ottawa Scale (NOS) adapted for cross-sectional studies; Figure S1: (A) Risk of bias summary: judgements about each bias item for each study, and (B) Risk of bias graph: review of authors’ judgements about each risk of bias item shown as percentages across all included studies.
