# Supplementary material for: Psoriasis and Vitamin D: A Systematic Review and Meta-Analysis
Source: Nutrients. 2023 Jul 30;15(15):3387. doi: 10.3390/nu15153387 (PMC10421389; doi:10.3390/nu15153387)

Supplementary materials

Supplementary Table S1. PRISMA 2020 for Abstracts checklist 2

Supplementary Table S2. PRISMA 2020 checklist 3

Supplementary Table S3. Search strategy of electronic databases 6

Supplementary Table S4. Complete dataset for the case-control and cross-sectional analysis 7

Supplementary Table S5. Complete dataset for the randomized controlled studies included in Vitamin D supplementation analysis 9

Supplementary Table S6. Quality scoring for included 18 articles using Newcastle-Ottawa Scale (NOS) for case-control studies 10

Supplementary Table S7. Quality scoring for included 5 articles using Newcastle-Ottawa Scale (NOS) adapted for cross-sectional studies 11

Supplementary Figure S1. A) Risk of bias summary: judgements about each bias item for each study, and B) Risk of bias graph: review authors' judgements about each risk of bias item showed as percentages across all included studies. 12

Supplementary Table S1. PRISMA 2020 for Abstracts checklist

Section and Topic	Item #	Checklist item	Reported (Yes/No)
**TITLE**			
Title	1	Identify the report as a systematic review.	Yes
**BACKGROUND**			
Objectives	2	Provide an explicit statement of the main objective(s) or question(s) the review addresses.	Yes
**METHODS**			
Eligibility criteria	3	Specify the inclusion and exclusion criteria for the review.	Yes
Information sources	4	Specify the information sources (e.g. databases, registers) used to identify studies and the date when each was last searched.	Yes
Risk of bias	5	Specify the methods used to assess risk of bias in the included studies.	Yes
Synthesis of results	6	Specify the methods used to present and synthesise results.	Yes
**RESULTS**			
Included studies	7	Give the total number of included studies and participants and summarise relevant characteristics of studies.	Yes
Synthesis of results	8	Present results for main outcomes, preferably indicating the number of included studies and participants for each. If meta-analysis was done, report the summary estimate and confidence/credible interval. If comparing groups, indicate the direction of the effect (i.e. which group is favoured).	Yes
**DISCUSSION**			
Limitations of evidence	9	Provide a brief summary of the limitations of the evidence included in the review (e.g. study risk of bias, inconsistency and imprecision).	Yes
Interpretation	10	Provide a general interpretation of the results and important implications.	Yes
**OTHER**			
Funding	11	Specify the primary source of funding for the review.	NA
Registration	12	Provide the register name and registration number.	NA

Supplementary Table S2. PRISMA 2020 checklist

Section and Topic	Item #	Checklist item	Location where item is reported
**TITLE**			
Title	1	Identify the report as a systematic review.	Lines 2-3
**ABSTRACT**			
Abstract	2	See the PRISMA 2020 for Abstracts checklist.	Done
**INTRODUCTION**			
Rationale	3	Describe the rationale for the review in the context of existing knowledge.	Lines 60-64
Objectives	4	Provide an explicit statement of the objective(s) or question(s) the review addresses.	Lines 65-68
**METHODS**			
Eligibility criteria	5	Specify the inclusion and exclusion criteria for the review and how studies were grouped for the syntheses.	Lines 74-95
Information sources	6	Specify all databases, registers, websites, organisations, reference lists and other sources searched or consulted to identify studies. Specify the date when each source was last searched or consulted.	Lines 96-104
Search strategy	7	Present the full search strategies for all databases, registers and websites, including any filters and limits used.	Lines 97-100 + Table S3
Selection process	8	Specify the methods used to decide whether a study met the inclusion criteria of the review, including how many reviewers screened each record and each report retrieved, whether they worked independently, and if applicable, details of automation tools used in the process.	Lines 100-104
Data collection process	9	Specify the methods used to collect data from reports, including how many reviewers collected data from each report, whether they worked independently, any processes for obtaining or confirming data from study investigators, and if applicable, details of automation tools used in the process.	Lines 105-118
Data items	10a	List and define all outcomes for which data were sought. Specify whether all results that were compatible with each outcome domain in each study were sought (e.g. for all measures, time points, analyses), and if not, the methods used to decide which results to collect.	Lines 105-118
	10b	List and define all other variables for which data were sought (e.g. participant and intervention characteristics, funding sources). Describe any assumptions made about any missing or unclear information.	Lines 105-109
Study risk of bias assessment	11	Specify the methods used to assess risk of bias in the included studies, including details of the tool(s) used, how many reviewers assessed each study and whether they worked independently, and if applicable, details of automation tools used in the process.	Lines 119-125
Effect measures	12	Specify for each outcome the effect measure(s) (e.g. risk ratio, mean difference) used in the synthesis or presentation of results.	Lines 128-129
Synthesis methods	13a	Describe the processes used to decide which studies were eligible for each synthesis (e.g. tabulating the study intervention characteristics and comparing against the planned groups for each synthesis (item #5)).	Lines 101-104
	13b	Describe any methods required to prepare the data for presentation or synthesis, such as handling of missing summary statistics, or data conversions.	Lines 126-139
	13c	Describe any methods used to tabulate or visually display results of individual studies and syntheses.	Lines 138-139

Section and Topic	Item #	Checklist item	Location where item is reported
	13d	Describe any methods used to synthesize results and provide a rationale for the choice(s). If meta-analysis was performed, describe the model(s), method(s) to identify the presence and extent of statistical heterogeneity, and software package(s) used.	Lines 126-139
	13e	Describe any methods used to explore possible causes of heterogeneity among study results (e.g. subgroup analysis, meta-regression).	Lines 126-139
	13f	Describe any sensitivity analyses conducted to assess robustness of the synthesized results.	Lines 135-139
Reporting bias assessment	14	Describe any methods used to assess risk of bias due to missing results in a synthesis (arising from reporting biases).	Lines 136-137
Certainty assessment	15	Describe any methods used to assess certainty (or confidence) in the body of evidence for an outcome.	Lines 119-125
**RESULTS**			
Study selection	16a	Describe the results of the search and selection process, from the number of records identified in the search to the number of studies included in the review, ideally using a flow diagram.	Figure 1
	16b	Cite studies that might appear to meet the inclusion criteria, but which were excluded, and explain why they were excluded.	Figure 1
Study characteristics	17	Cite each included study and present its characteristics.	Table 1 and Table 2
Risk of bias in studies	18	Present assessments of risk of bias for each included study.	Table S6, S7, Figure S1
Results of individual studies	19	For all outcomes, present, for each study: (a) summary statistics for each group (where appropriate) and (b) an effect estimate and its precision (e.g. confidence/credible interval), ideally using structured tables or plots.	Lines 148-213
Results of syntheses	20a	For each synthesis, briefly summarise the characteristics and risk of bias among contributing studies.	Lines 217-221
	20b	Present results of all statistical syntheses conducted. If meta-analysis was done, present for each the summary estimate and its precision (e.g. confidence/credible interval) and measures of statistical heterogeneity. If comparing groups, describe the direction of the effect.	Lines 148-213
	20c	Present results of all investigations of possible causes of heterogeneity among study results.	Lines 148-213
	20d	Present results of all sensitivity analyses conducted to assess the robustness of the synthesized results.	Lines 148-213
Reporting biases	21	Present assessments of risk of bias due to missing results (arising from reporting biases) for each synthesis assessed.	Lines 148-221
Certainty of evidence	22	Present assessments of certainty (or confidence) in the body of evidence for each outcome assessed.	Lines 148-213
**DISCUSSION**			
Discussion	23a	Provide a general interpretation of the results in the context of other evidence.	Lines 230-237 and 298-331
	23b	Discuss any limitations of the evidence included in the review.	Lines 340-345
	23c	Discuss any limitations of the review processes used.	Lines 334-345

Section and Topic	Item #	Checklist item	Location where item is reported
	23d	Discuss implications of the results for practice, policy, and future research.	Lines 346-360
**OTHER INFORMATION**			
Registration and protocol	24a	Provide registration information for the review, including register name and registration number, or state that the review was not registered.	NA
	24b	Indicate where the review protocol can be accessed, or state that a protocol was not prepared.	NA
	24c	Describe and explain any amendments to information provided at registration or in the protocol.	NA
Support	25	Describe sources of financial or non-financial support for the review, and the role of the funders or sponsors in the review.	Line 374
Competing interests	26	Declare any competing interests of review authors.	Line 375
Availability of data, code and other materials	27	Report which of the following are publicly available and where they can be found: template data collection forms; data extracted from included studies; data used for all analyses; analytic code; any other materials used in the review.	Lines 569-571

Supplementary Table S3. Search strategy of electronic databases

Books@Ovid
Journals@Ovid Full Text <July 08, 2023>
Your Journals@Ovid
Biological Abstracts <2022>
Biological Abstracts <2018 to 2021>
Biological Abstracts <2017>
Biological Abstracts <2016>
Biological Abstracts <2015>
Biological Abstracts <2014>
Econlit <1886 to June 29, 2023>
Global Health <1973 to 2023 Week 26>
International Political Science Abstract <1989 to April 2023>
Ovid MEDLINE(R) ALL <1946 to July 11, 2023>
The Philosopher's Index <1940 to May 2023>

1. ((vitamin D or vitamin D2 or vitamin D3 or D2 or D3 or ergocalciferol or cholecalciferol or 25-hydroxyvitamin D) and psoriasis).mp. [mp=tx, bt, bo, ti, ab, ct, mc, st, or, tn, ps, ds, cb, rn, sq, mq, ge, tm, mi, sh, hw, ot, cw, an, ui, jn, tt, nm, fx, kf, ox, px, rx, on, sy, ux, mx] 7,963
2. "query=vitamin D OR vitamin D2 OR vitamin D3 OR D2 OR D3 OR ergocalciferol OR cholecalciferol OR 25-hydroxyvitamin D AND psoriasis","desiredResults=10000","minHitsDivisor=7","permitHyponyms=NO","lowestVocabularySearchLevel=none","phrasesBroken=NO","speedWanted=Fastest","comment=No Related Terms","elimEnable=NO","constraintMinTerms=2" 6031

PubMed Advanced Search Builder

Search: (vitamin D OR vitamin D2 OR vitamin D3 OR D2 OR D3 OR ergocalciferol OR cholecalciferol OR 25-hydroxyvitamin D) AND (psoriasis)
Sort by: Most Recent
("vitamin d"[MeSH Terms] OR "vitamin d"[All Fields] OR "ergocalciferols"[MeSH Terms] OR "ergocalciferols"[All Fields] OR ("ergocalciferols"[MeSH Terms] OR "ergocalciferols"[All Fields] OR ("vitamin"[All Fields] AND "D2"[All Fields]) OR "vitamin d2"[All Fields]) OR ("cholecalciferol"[MeSH Terms] OR "cholecalciferol"[All Fields] OR ("vitamin"[All Fields] AND "D3"[All Fields]) OR "vitamin d3"[All Fields]) OR "D2"[All Fields] OR "D3"[All Fields] OR ("ergocalciferols"[MeSH Terms] OR "ergocalciferols"[All Fields] OR "ergocalciferol"[All Fields]) OR ("cholecalciferol"[MeSH Terms] OR "cholecalciferol"[All Fields] OR "cholecalciferols"[All Fields] OR "colecalciferol"[All Fields]) OR ("25 hydroxyvitamin d"[Supplementary Concept] OR "25 hydroxyvitamin d"[All Fields] OR "25 hydroxyvitamin d"[All Fields] OR "calcifediol"[MeSH Terms] OR "calcifediol"[All Fields])) AND ("psoriasis"[MeSH Terms] OR "psoriasis"[All Fields] OR "psoriases"[All Fields] OR "psoriasi"[All Fields])

Supplementary Table S4. Complete dataset for the case-control and cross-sectional analysis

Paper	N	Psoriasis patients	Controls	Serum 25(OH)D levels of psoriasis patients	Serum 25(OH)D levels of controls	Serum calcium levels of psoriasis patients	Serum calcium levels of controls	Serum phosphorus levels of psoriasis patients	Serum phosphorus levels of controls	Serum PTH levels of psoriasis patients	Serum PTH levels of controls
Bhat GH; 2022 [27]	602	285	317	28.3±15.0	37.8±9.8						
Pokharel R; 2022 [28]	180	120	60	19.6±6.9	23.6±6.4						
Patil A; 2022 [29]	84	42	42	18.7±7.8	22.7±7.7						
Chandrashekar L; 2015 [30]	86	43	43	13.3±6.9	22.4±18.4						
Zuchi MF; 2015 [31]	40	20	20	23.6±7.6	22.4±3.1						
Petho; 2015 [32]	106	53	53	20.7±7.1	26.9±11.1	9.6±0.4	9.2±0.4			41.5±14.9	35.0±10.1
Orgaz-Molina J; 2014 [33]	92	46	46	30.5±9.3	38.3±9.6	9.3±0.4	9.3±0.4	3.3±0.5	3.1±0.6	41.9±14.7	39.8±14.1
Solak B; 2016 [34]	84	43	41	21.2±8.7	25.2±14.1	9.4±0.4	9.7±0.2	3.3±0.5	3.4±0.6	84.2±39.0	51.4±24.2
Al-Mutairi N; 2013 [35]	200	100	100	31.5±14.4	53.5±19.6						
Filoni A; 2021 [36]	96	48	48	21.7±10.4	25.6±7.4	9.7±0.4	9.6±0.4	3.6±0.4	3.7±0.7	24.3±10.5	22.1±7.9
Flioni A; 2018 [37]	510	170	340	21.8±12.3	34.3±13.3						
Staberg B; 1987 [38]	69	32	37	22.9±16.5	27.8±12.8						
Hata TR; 2014 [39]	46	16	30	29.8 ±9.5	30.1 ±12.4						

Kuang Y; 2020 [40]	408	203	205	9.6±3.7	9.5±3.1						
Nayak PB; 2018 [41]	122	61	61	18.4±9.4	17.2±13.1	9.7±0.6	9.6±0.6				
Orgaz-Molina J; 2012 [42]	86	43	43	24.4±7.8	29.5±9.4						
Bergler-Czop B; 2016 [43]	80	40	40	12.9±2.7	22.5±2.7						
Alhetheli G; 2022 [44]	94	53	41	22.5±4.9	38.9±5.4						
Atwa M; 2013 [45]	83	43	40	11.7±3.6	24.6±11.2					68.2±19.5	60.6±14.1
Maleki M; 2016 [46]	93	50	43	14.9±6.3	12.5±4.5						
Wilson PB; 2013 [47]	5841	148	5693	24.2±2.2	23.6±1.3						
Gisondi P; 2011 [48]	286	145	141	20.7±11.3	37.1±27.6	9.5±0.4	9.3±0.3			58.7±24.5	56.0±18.9
Grassi T; 2020 [49]	120	72	48	23.1±10.1	25.3±10.2	9.7±0.6	9.6±0.6	3.6±0.6	3.7±0.6	22.5±8.7	22.6±8.8

Abbreviations: 25(OH)D: 25 hydroxyvitamin D, PTH: parathyroid hormone.

Supplementary Table S5. Complete dataset for the randomized controlled studies included in Vitamin D supplementation analysis

Paper	N	Vitamin D supplementation group	Placebo group	3-months PASI		6-months PASI		12-months PASI	
				Vitamin D supplementation group	Control group	Vitamin D supplementation group	Control group	Vitamin D supplementation group	Control group
Ingram M; 2018 [50]	101	67	34	4.5±3.3	4.8±3.0	4.5±4.6	4.7±3.3	3.6±1.9	4.1±3.3
Disphanurat W; 2019 [51]	45	23	22	4.7±3.9	3.1±2.4	3.4±2.5	2.4±2.0		
Jarrett P; 2018 [52]	65	23	42	2.5±1.2	2.3±1.7	2.5±1.2	2.1±1.7	2.21.2	2.1±1.7
Jenssen M; 2023* [53]	122	60	62	2.9±1.9	2.6±1.7				

*Follow-up data are available only at 4 months.

Abbreviations: PASI: Psoriasis Area and Severity Index.

Supplementary Table S6. Quality scoring for included 18 articles using Newcastle-Ottawa Scale (NOS) for case-control studies

**Reference**	Case definition	Case representation	Control selection	Control definition	Comparability	Exposure ascertainment	Same method of ascertainment	Non-response rate	Total*
Bhat GH; 2022 [27]	A*	A*	A*	A*	A*	A*	A*	A*	8
Pokharel R; 2022 [28]	A*	A*	A*	A*	A*	A*	A*	A*	8
Patil A; 2022 [29]	A*	A*	C	A*	A*B*	A*	A*	A*	8
Chandrashekar L; 2015 [30]	A*	A*	C	A*	A*B*	A*	A*	A*	8
Zuchi MF; 2014 [31]	A*	A*	B	A*	A*	A*	A*	A*	7
Petho Z; 2015 [32]	A*	A*	A*	A*	A*B*	A*	A*	A*	9
Orgaz-Molina J; 2014 [33]	A*	A*	B	A*	A*B*	A*	A*	A*	8
Solak B; 2016 [34]	A*	A*	A*	A*	A*B*	A*	A*	A*	9
Al-Mutairi N; 2013 [35]	A*	A*	A*	A*	A*	A*	A*	A*	8
Filoni A; 2021 [36]	A*	A*	A*	A*	A*B*	A*	A*	A*	9
Filoni A; 2018 [37]	A*	A*	B	A*	A*	A*	A*	A*	7
Staberg B; 1987 [38]	A*	A*	C	A*	A*	A*	A*	A*	7
Hata TR; 2014 † [39]	A*	A*	C	A*	A*	A*	A*	A*	7
Kuang Y; 2020 [40]	A*	A*	A*	A*	A*	A*	A*	A*	8
Nayak PB; 2018 [41]	A*	A*	B	A*	A*B*	A*	A*	A*	8
Orgaz-Molina J; 2012 [42]	A*	A*	B	A*	A*	A*	A*	A*	7

Bergler-Czop B; 2016 [43]	A*	A*	C	A*	A*	A*	A*	A*	7
Alhetheli G; 2022 [44]	A*	A*	B	A*	A*	A*	A*	A*	7

†The study by Hata et al. is reported as a case-control study since we considered baseline values.

Supplementary Table S7. Quality scoring for included 5 articles using Newcastle-Ottawa Scale (NOS) adapted for cross-sectional studies

**Reference**	Representativeness of the cases	Sample size	Non-response rate	Ascertainment of the exposure	Comparability	Assessment of the outcome	Statistical test	Total*
Atwa M; 2013 [45]	B*	A*	C	A**	A*	C*	A*	7
Maleki M; 2016 [46]	B*	A*	C	A**	A*B *	C*	A*	8
Wilson PB; 2013 [47]	B*	A*	C	A**	A*	C*	A*	7
Gisondi P; 2011 [48]	B*	A*	C	A**	A*B *	C*	A*	8
Grassi T; 2020 [49]	B*	A*	C	A**	A*B *	C*	A*	8

Supplementary Figure S1. A) Risk of bias summary: judgements about each bias item for each study [53, 52, 50, 51], and B) Risk of bias graph: review authors' judgements about each risk of bias item showed as percentages across all included studies.

A)

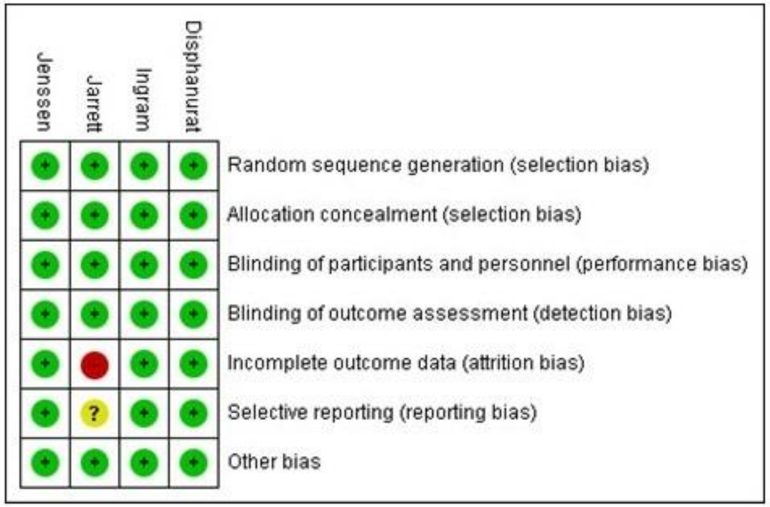

B)

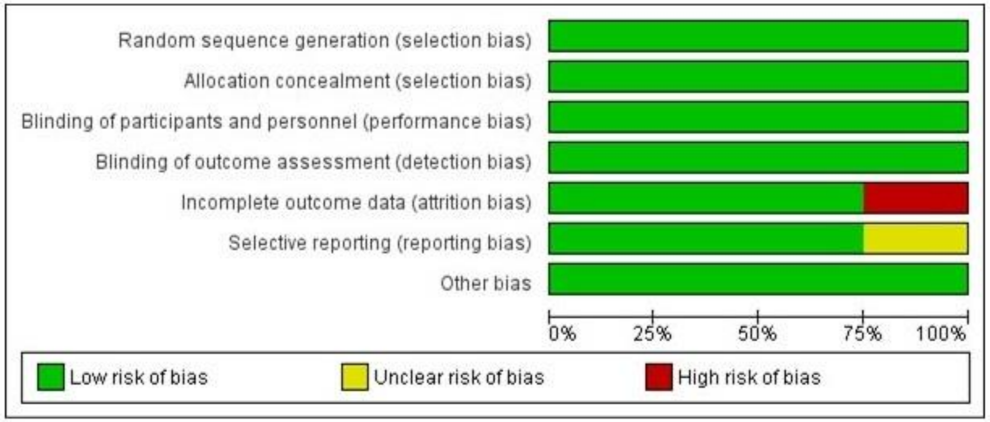

Supplement: Supplementary file 1 [file nutrients-15-03387-s001.zip › nutrients-2494215-supplementary.pdf]
